# Mapping genomic regions associated with temperature stress in the wheat pathogen *Zymoseptoria tritici*

**DOI:** 10.1093/g3journal/jkaf094

**Published:** 2025-04-26

**Authors:** Jessica Stapley, Ziming Zhong, Bruce A McDonald

**Affiliations:** Plant Pathology Group, Institute of Integrative Biology, ETH Zurich, 8092 Zürich, Switzerland; Plant Pathology Group, Institute of Integrative Biology, ETH Zurich, 8092 Zürich, Switzerland; Plant Pathology Group, Institute of Integrative Biology, ETH Zurich, 8092 Zürich, Switzerland

**Keywords:** heat stress, plant pathogen, fungi, *Mycosphaerella graminicola*, QTL, quantitative trait loci

## Abstract

Climate change can alter interactions between plants and their pathogens, which could adversely affect crop production. To better understand the molecular mechanisms underlying the responses of pathogenic fungi to temperature stress, we conducted a quantitative trait loci (QTL) mapping study in the wheat pathogen *Zymoseptoria tritici* to identify genomic regions associated with colony growth and melanization at 3 temperatures (10, 18, and 27°C). We then identified likely candidate genes for thermal adaptation within these intervals by combining information regarding gene function, gene ontology (GO) annotation enrichment, transcriptional profile, and results from previous genome-wide association studies investigating responses to climate, temperature, and thermal adaptation. The QTL mapping, conducted for 2 separate crosses involving 4 Swiss parents, found significant QTL uniquely associated with traits measured in high and low temperatures. These intervals contained many genes known to regulate responses to temperature stress, including heat-shock proteins and proteins involved in the mitogen-activated protein kinase (MAPK) pathways, and were enriched for genes with a zinc ion binding GO annotation. We highlight the most promising candidate genes for thermal adaptation, including an ammonium transporter gene, a stress response factor (*Whi1*) and 2 MAPK pathway genes—*SSk2* and *Opy2*. Future validation work on these candidate genes could provide novel insight into the molecular mechanisms underlying temperature adaptation in this important wheat pathogen.

## Introduction

Human-induced climate change is the greatest challenge facing our planet. The warming climate will affect crop production and may jeopardize food security. One important consequence of climate change in this respect is that it is likely to alter interactions between plants and their pathogens (Chaloner *et al*. 2021; Qiu *et al*. 2022; Raza and Bebber 2022; Singh *et al*. 2023). A recent review of 45 studies on model crop species found that in 55% of cases, disease resistance was negatively affected by elevated temperature (Desaint *et al*. 2021). This may be driven by changes within the plant, e.g. warmer temperatures may compromise plant basal resistance (Qiu *et al*. 2022), or by changes within the pathogen, e.g. elevated temperatures can increase pathogen growth rates and reproduction (Vaumourin and Laine 2018). Pathogen–plant interactions can also be altered due to shared molecular mechanisms underlying virulence and heat tolerance. For example, in rice blast (*Pyricularia oryzae*), genes involved in virulence also influence heat tolerance (Li *et al*. 2011). In addition, temperature can alter the sensitivity of plant pathogens to fungicides (Lurwanu *et al*. 2021). Predicting how these interactions will change requires a detailed understanding of how plants and their pathogens respond to changes in temperature as well as knowledge of the molecular mechanisms underlying their response to temperature stress. Increased knowledge of the genes involved in a pathogen’s response to temperature stress may also provide insights into the vulnerabilities of pathogens and potentially identify novel targets for control.

Several molecular responses to temperature stress, particularly higher temperatures, have been well characterized in fungi (e.g. Richter *et al*. 2010; Dunayevich *et al*. 2018; Bakar *et al*. 2020). In fungi, as in all eukaryotes, small increases in temperature can cause changes in the 3D structure of enzymes and proteins, including unfolding, entanglement, and nonspecific aggregation of proteins, and can also affect RNA splicing, DNA replication, cellular membranes, and the cytoskeleton (Richter *et al*. 2010). Repairs to the functional structures of proteins to maintain cell homeostasis are achieved by interactions with molecular chaperones, often referred to as heat-shock proteins (HSPs), that prevent aggregation of misfolded proteins and promote efficient protein folding. Although HSPs were first discovered in studies of response to heat shock, these molecular chaperones play key roles in the general stress response, and the initiation of the heat-shock response is not necessarily due to changes in temperature but rather due to the detection of unfolded proteins (Richter *et al*. 2010; Zheng *et al*. 2016).

Temperature stress response pathways can overlap with pathways associated with osmotic and oxidative stress. Heat stress can increase damage from oxidative stress caused by the accumulation of reactive oxygen species, and induce osmotic stress caused by osmotic pressure changes (Xiao *et al*. 2022). The mitogen-activated protein kinase (MAPK) pathways are important in mediating responses to temperature, oxidative, and osmotic stress in fungi. In yeast, there are 5 MAPK pathways, 2 of which are activated and interact under temperature stress, including (1) the high-osmolarity glycerol (HOG) pathway (Winkler *et al*. 2002; Dunayevich *et al*. 2018) and (2) the cell-wall integrity (CWI) pathway (Sanz *et al*. 2017). In yeast, heat stress triggers the release of glycerol by activation of CWI; this causes a loss of turgor pressure, which stimulates the HOG1 pathway (Dunayevich *et al*. 2018). In *Zymoseptoria tritici*, *Pbs2*, a HOG1 pathway gene, was identified as a possible candidate gene for thermal sensitivity (Lendenmann *et al*. 2016), and heat stress has been shown to trigger intracellular osmotic stress, which triggers the CWI and the HOG pathway and results in hyphal growth (Francisco *et al*. 2023). These studies demonstrate that both molecular chaperones and MAPKs are integral to fungal response to temperature.

Here, we use quantitative trait loci (QTL) mapping in *Z. tritici* to identify genomic regions associated with responses to high and low temperature stress, enabling a search for candidate genes associated with thermal adaptation. In *Z. tritici*, QTL mapping, genome-wide association studies (GWASs) and transcriptomic studies have already made important contributions to our understanding of the molecular mechanisms underlying fungal response and adaptation to temperature. One of the first QTL studies in *Z. tritici* investigated how colony growth is affected by temperatures that are cooler (15°C) or warmer (22°C) than the optimal growth temperature. This study identified a cool temperature sensitivity QTL on chromosome 10 that contained the candidate gene *Pbs2*, a MAPKK involved in the HOG pathway (Lendenmann *et al*. 2016). Two GWAS studies analyzed SNP associations using phenotypic data acquired at different temperatures. The first GWAS used 145 global strains to identify many significant SNPs associated with colony size and melanization at 15 and 22°C, hereafter referred to as the trait-based GWAS or “traitGWAS” (Dutta *et al*. 2023). The second used a larger global panel of 411 strains and measured growth traits in liquid media across a broader temperature range (12, 15, 18, 22, and 25°C) to identify SNPs and candidate genes associated with thermal adaptation, hereafter called “adaptGWAS” (Miñana-Posada *et al*. 2025). A third GWAS study used a genotype-by-environment association approach (hereafter called “geaGWAS”), based on bioclimatic data obtained from the sampling locations of over 1,000 global strains (Feurtey *et al*. 2023). This study found many significant SNP associations and highlighted promising candidate genes for temperature adaptation.

### Aims

The aim of this study was to identify large effect loci associated with responses to high (27°C) and low (10°C) temperatures in *Z. tritici* and to identify possible candidate genes within the identified QTL intervals. The QTL study reported here extends the previous QTL and GWAS work by including a broader range of temperatures (10–27°C) and using a reference genome for one of the parents used in each cross instead of the previous IPO323 reference genome. This new QTL study is distinct from the GWAS studies that used a genotype-by-environment association approach, as we experimentally manipulated growth temperatures. To find the most promising candidate genes to include in future functional validation experiments, we first focused on the QTL intervals that were unique to temperature stress environments (10 and 27°C), excluding QTL associated with the same traits measured in the benign temperature environment (18°C). We then used information about gene function, paying attention to known temperature-response genes (Temp-genes, Supplementary Table 1), gene ontology (GO) enrichment, gene variation between the parents, and genes identified in the earlier GWAS analyses to identify the most promising candidate genes.

## Methods

### QTL mapping crosses

Parent and progeny isolates used in this study are from 2 crosses between Swiss strains that were described previously (Lendenmann *et al*. 2014). The original choice of strains to cross was based upon 7 measured traits for each strain, including in planta virulence, pycnidia size and density, and in vitro growth rates under different temperatures and in the presence of fungicides (Zhan *et al*. 2005). The 2 crosses yielded 700 offspring to use for QTL mapping. The 4 parents were later phenotyped under other environmental stressors (e.g. reactive oxygen; Zhong *et al*. 2021) and at 2 temperatures (15 and 22°C) (Lendenmann *et al*. 2016). The cross between ST99CH1A5 and ST99CH1E4 (herein referred to as 1A5 and 1E4, respectively) produced 341 progeny. The cross between ST99CH3D7 and ST99CH3D1 (herein referred to as 3D7 and 3D1, respectively) produced 359 progeny.

### Genotyping

SNP genotype data for each set of progeny were obtained from a RAD sequence dataset previously produced in our lab (first described in Lendenmann *et al*. 2014). In brief, the genome was cut using the restriction enzyme *Pst*l, and the libraries were sequenced on an Illumina HiSeq2000 with paired-end sequencing. Complete genome sequences of the parental strains [NCBI Biosample: SRS383146 (ST99CH3D1), SRS383147 (ST99CH3D7), SRS383142 (ST99CH1A5), and SRS383143 (ST99CH1E4)] were used to SNP genotype the parents and offspring (Croll *et al*. 2013).

RADseq processing, variant discovery, and linkage maps are described in detail at https://github.com/jessstapley/QTL-mapping-Z.-tritici and in an earlier publication (Stapley and McDonald 2023). In brief, RADseq reads were trimmed of adapters and low-quality sequences using trimmomatic (v0.35). The RADseq reads were mapped to a reference genome using bwa mem (v0.7.17). Reads from progeny of the 3D7 × 3D1 cross were mapped to the 3D7 reference genome and progeny from the 1A5 × 1E4 cross were mapped to the 1A5 reference genome. Variant calling was done using the GATK Germline Short Variant Discovery pipeline, following their Best Practice recommendations (https://gatk.broadinstitute.org/hc/en-us/sections/360007226651-Best-Practices-Workflows). After this, we applied the following filters: only biallelic SNPs, parents had alternative alleles, <50% missing genotypes per marker, <50% missing genotypes per individual, mean read depth >3 and <30, depth quality >5, mapping quality >40, minor allele frequency >0.02 and <0.80, and SNPs in regions where the SNP density is >3 in 10 bp were removed. Linkage maps built using these SNPs were described previously (Stapley and McDonald 2023). After filtering and map building, 63,181 SNPs were available for analyzing the 3D7 × 3D1 cross and 32,806 SNPs were available for the 1A5 × 1E4 cross. A summary of the 2 linkage maps is provided in Supplementary Table 2, and the complete maps are available at https://github.com/jessstapley/QTL-mapping-Z.-tritici.

### Phenotyping

We compared in vitro colony growth and melanization at 18°C (control/benign environment) to that under cold stress (10°C) and heat stress (27°C). The optimal temperature for *Z. tritici* growing in vitro is 20.3°C (*T*_min_ = 4.9, *T*_max_ = 33.5; Chaloner *et al*. 2021). Only nonclonal offspring strains passing all filters were phenotyped (for 1A5 × 1E4 *n* = 259 strains; for 3D7 × 3D1 *n* = 265 strains). The protocols for isolate recovery from −80°C storage, growth in vitro, and the measurements of colony size and colony melanization were described previously (Zhong *et al*. 2021). In brief, Petri dishes containing potato dextrose agar were inoculated with 200 μL of a spore solution (concentration 200 spores/mL) and grown at 18°C for 8 and 12 days. Three replicates were grown at all temperatures. Two traits were measured; colony area and colony gray value, using automated image analysis, as described previously (Lendenmann *et al*. 2014; Zhong *et al*. 2021). Gray value was measured on a scale of 0–255, where darker, more melanized colonies have lower values (0 = black, 255 = white). Mean colony area and gray value were calculated from measurements of multiple colonies on each plate, and then, the mean was calculated across the 3 replicate plates to obtain a final mean colony area and mean gray value for each strain in the 2 environments. Colony area was converted to a radial size measure by dividing by *π* and taking the square root of this value.

As colony growth varied with temperature and cross, it was not possible to make all measurements at the same days postinoculation (dpi) in both crosses (see Table 1). At 10°C, colonies were measured at 12 and 15 dpi; at 18°C (control), colonies were measured at 8 and 12 dpi; and at 27°C, colonies were measured at 12 and 15 dpi in the 1A5 × 1E4 cross and at 8 and 12 dpi in the 3D1 × 3D7 cross. In addition, at 27°C, there was no colony growth for the 1E4 parent and 43% of the offspring in the 1A5 × 1E4 cross did not grow. Thus, all measurements at 27°C in the 1A5 × 1E4 cross had a much smaller sample size (Table 1).

**Table 1.** Number of isolates for each trait in each cross used for QTL mapping.

		Colony radius/gray value			Radius/gray value tolerance			Growth/melanization rate				Growth/melanization rate tolerance		
		Temperature				Cold	Heat		Temperature				Cold	Heat
	dpi	10°C	18°C	27°C	dpi	10–18°C	18–27°C	Interval	10°C	18°C	27°C	Interval	10–18°C	18–27°C
1A5 × 1E4	8	NA	251	NA	8	NA	NA	8–12	NA	249	NA	8–12	NA	NA
	12	243	250	141	12	243	141	12–15	218	NA	127	12–15	NA	NA
	15	225	NA	149	15	NA	NA							
3D7 × 3D1	8	NA	261	202	8	NA	198	8–12	NA	258	199	8–12	NA	194
	12	263	261	258	12	259	254	12–15	263	NA	256	12–15	NA	NA
	15	264	NA	258	15	NA	NA							

Colony radius and gray value were measured at multiple time points (dpi). Growth rate and melanization rate were calculated as the change in colony radius or gray value between subsequent time intervals (8–12, 12–15 dpi). Tolerance was estimated as the ratio between the trait value measured in the stressful temperature (10 and 27°C) and the control temperature (18°C), for either traits measured at specific time points (colony radius, gray value) or the rate measurements (colony growth, melanization). Colony measurements were not possible at all temperatures and time points because colonies grew at different rates and individual colonies could not be clearly distinguished on the plates. Thus, there are no data for colonies at 10°C 8 dpi and at 18°C at 15 dpi for both crosses, and no data for colonies at 27°C 8 dpi for the 1A5 × 1E4 cross. This also resulted in missing data for the corresponding rate and tolerance measurements, e.g. no growth/melanization rates at 10°C between 8 and 12 dpi, and no rate tolerance measurements for the 1A5 × 1E4 cross.

For the analysis, we used 3 different types of measurements; measurements at a single time point (dpi) representing a specific colony age: including the colony radius and gray value at 8, 12, and 15 dpi; daily rate measurements at each temperature: the change in colony radius or gray value between ages: 8–12 or 12–15 dpi (e.g. the difference in colony radius between 8 and 12 dpi/difference in days); and tolerance measurements: the ratio of a measurement (colony radius, gray value, growth rate, or melanization rate) between a stressful temperature environment and the control environment at the same dpi, e.g. radius at 8 dpi at 27°C/radius at 8 dpi at 18°C, or across the same interval, e.g. growth rate at 27°C/growth rate at 18°C. For the tolerance measurements, values >1 indicate the isolate was more tolerant to temperature stress (grew faster under temperature stress), and values <1 indicate that the isolate was more sensitive to temperature stress. As stated above, only 43% of the offspring from the 1A5 × 1E4 cross grew at 27°C. To consider survival at 27°C as a trait in our QTL analysis, we calculated a binary trait value—colony growth or no colony growth—and searched for QTL associated with this binary trait using a binary model. In total, there are 30 traits in the 3D7 × 3D1 cross and 24 traits in the 1A5 × 1E4 cross (Table 2).

**Table 2.** Summary of the QTL mapping results for all traits.

Trait full name	QTL 3D7 × 3D1	*h* ^2^	QTL 1A5 × 1E4	*h* ^2^
Colony radius in 10°C at 12 dpi	6	0.30	2,8	0.55
Colony radius in 10°C at 15 dpi	8,10	0.39	2,8	0.43
Colony radius in 18°C at 8 dpi	3,8,10	0.53	2,3,8	0.56
Colony radius in 18°C at 12 dpi	3,7,8,10,11	0.62	7,8	0.48
Colony radius in 27°C at 8 dpi	1,4	0.58	NA	NA
Colony radius in 27°C at 12 dpi	1,4	0.42	3	0.33
Colony radius in 27°C at 15 dpi	1,3	0.36	2	0.43
Gray value in 10°C at 12 dpi	8,10	0.55	2,8,12	0.48
Gray value in 10°C at 15 dpi	8,10,11	0.51	2,8,12	0.47
Gray value in 18°C at 8 dpi	8,10,11	0.42	1,2,3,8	0.63
Gray value in 18°C at 12 dpi	11	0.22	2,8	0.44
Gray value in 27°C at 8 dpi	11	0.81	NA	NA
Gray value in 27°C at 12 dpi	1,11	0.64	No QTL	0.40
Gray value in 27°C at 15 dpi	1,3,11	0.56	No QTL	0.22
Growth rate 10°C 12–15 dpi	10	0.40	No QTL	0.28
Growth rate 18°C 8–12 dpi	3,8,11	0.61	7,8	0.33
Growth rate 27°C 8–12 dpi	1,3	0.33	NA	NA
Growth rate 27°C 12–15 dpi	1	0.21	2	0.30
Melanization rate 10°C 12–15 dpi	8,10	0.35	2,8,12	0.47
Melanization rate 18°C 8–12 dpi	10	0.28	2,3	0.54
Melanization rate 27°C 8–12 dpi	11	0.26	NA	NA
Melanization rate 27°C 12–15 dpi	3,11	0.31	No QTL	0.28
Colony radius cold tolerance at 12 dpi	3,11	0.44	2	0.33
Colony radius heat tolerance at 8 dpi	1,5,10	0.60	NA	NA
Colony radius heat tolerance at 12 dpi	1,5,8,10,11	0.57	3	0.35
Gray value cold tolerance at 12 dpi	11	0.22	3	0.40
Gray value heat tolerance at 8 dpi	11	0.43	NA	NA
Gray value heat tolerance at 12 dpi	11	0.36	8	0.39
Growth rate heat tolerance at 8–12 dpi	No QTL	0.48	NA	NA
Melanization rate heat tolerance at 8–12 dpi	8,11	0.26	NA	NA
Survival (colony presence/absence) in 27°C at 12 dpi	NA	NA	1	0.48

Details for each trait are described in Table 1. The QTL columns indicate the chromosomes on which significant QTL peaks were found for each trait in the 3D7 × 3D1 and 1A5 × 1E4 crosses. The *h*^2^ columns show the estimated heritability (*h*^2^) for each trait in each cross.

Differences in sampling dpi for stressful and benign temperatures limited our ability to compare traits between benign and stressful environments. We could only compare colony radius/gray value between 10 and 18°C at 12 dpi, 18 and 27°C at 12 dpi in the 1A5 × 1E4 cross, and 18 and 27°C at 8 and 12 dpi in the 3D7 × 3D1 cross (Table 1). Radial size and gray value tolerance could only be calculated at 12 dpi in both crosses, and heat tolerance at 8 dpi in the 3D7 × 3D1 cross. For growth rate and melanization rate tolerance, only heat tolerance (27°C compared with 18°C) could be calculated in the 3D7 × 3D1 cross, because growth rate and melanization rate were measured over the same interval (8–12 dpi) at 27 and 18°C. The same tolerance values are not available for the 1A5 × 1E4 cross or for colonies grown at 10°C, because the colonies were measured at different times between 18 and 10°C (Table 1).

### QTL mapping

QTL mapping was performed using the R (v 3.6.0) package “qtl2” (v2_0.24), as described in detail on github (https://github.com/jessstapley/QTL-mapping-Z.-tritici). We scanned the genome for a single QTL per chromosome with the “*scan1*” function using a linear mixed effect model. For continuous traits, we fitted the kinship matrix in the model to control for the relatedness of individuals (i.e. we included a random polygenic effect). Models that take into account the genetic covariance between individuals can reduce the false discovery rate in QTL scans and outperform models that do not include this information in the model (Malosetti *et al*. 2011). For the binary trait, we fitted a binary model, without the kinship matrix as this is not possible with this R package. The significance threshold for a QTL peak was determined by 1,000 permutation tests, and we calculated a Bayes credible interval (95%) to identify the interval size around the QTL peak. Overlapping QTLs were grouped to identify regions uniquely associated with traits in stressful and benign temperatures. QTLs were placed in the same group if the peak marker of 1 QTL interval overlapped with the 95% Bayes credible interval of another QTL.

### Identifying putative candidate genes in unique temperature stress QTL

A QTL uniquely associated with temperature stress is one where the peak marker for a QTL associated with temperature stress (10 or 27°C) did not fall within the 95% credible interval of the QTL interval associated with a trait measured at 18°C. We focus on these unique temperature stress QTL, as these provide the best opportunity to find genes specifically related to temperature response. Within each unique temperature stress QTL interval, we gathered information about each gene in both parents to identify the most likely candidate genes associated with temperature stress. This gene information included the predicted gene function—paying particular attention to genes with known roles in temperature stress response (Temp-genes, Supplementary Table 1), sequence (gene and amino acid) variation between the parents, expression differences between parents in vitro and in vivo, GO enrichment, and whether the gene contained SNPs that were previously found to be associated with climate variables in a previous global environmental GWAS (Feurtey *et al*. 2023).

### Gene orthologs, function, and variation in parents

We used custom R scripts to extract the list of genes within the interval from the annotation (gff) files of each reference genome. The putative encoded function of each gene was determined in previous analyses (for 1A5, see Plissonneau *et al*. 2018, and for 3D7, see Plissonneau *et al*. 2016). The annotations were obtained using InterProScan (1A5: v5.16-55.0, and 3D7: v5.18-57; https://www.ebi.ac.uk/interpro/) against multiple protein databases. Protein sequences were also screened for secretion signals, cytoplasmic, transmembrane, and extracellular domains using multiple methods (SignalP v4.1 https://services.healthtech.dtu.dk/service.php?SignalP-5.0, Phobius v1.01 https://phobius.sbc.su.se/, and TMHMM 2.0 http://www.cbs.dtu.dk/services/TMHMM/). The putative effect of an SNP in the coding sequence was determined using SNPeff (http://pcingola.github.io/SnpEff/). We identified the orthologous genes in the nonreference parent (3D7, 1E4) using an analysis performed across the genomes of 19 reference strains (Badet *et al*. 2020). We compared the DNA sequence and amino acid similarity between parents. DNA sequences were compared with BLAST, and amino acid sequences were aligned and compared using R:protr (Xiao *et al*. 2015).

### RNAseq data analysis

We analyzed RNAseq data that were previously created for the 4 parental strains during infection of the susceptible wheat cultivar Drifter (Palma-Guerrero *et al*. 2016, 2017) as well as cultures grown in vitro (Francisco *et al*. 2019). For in planta infection, Drifter seedlings were inoculated with the 4 strains. The second leaf on 10 different plants was harvested at 3, 7, 10, 12, 14, 16, 18, 20, 24, and 28 dpi for each strain. Total RNA was extracted from infected leaves at 4 time points (7, 12, 14, and 28 dpi) using TRIzol (Invitrogen), following the manufacturer's recommendations. The 3 leaf samples for each time point were used as biological replicates for library preparation and sequencing. For in vitro datasets, 2 types of liquid media were used: yeast sucrose broth (YSB): yeast extract 10 g/L and sucrose 10 g/L, pH 6.8; and carbon-depleted minimal medium (MM), pH 5.8 (Francisco *et al*. 2019). Flasks containing a final concentration of 105 blastospores/mL in YSB or C-depleted medium were incubated for 4 days at 18°C to induce blastosporulation or mycelial growth, respectively. Total RNA was extracted from 3 replicates per strain and condition by using TRIzol (Thermo Fisher Scientific, Waltham, MA, USA) following the manufacturer's instructions. The analysis was described previously (Stapley and McDonald 2023). The raw sequence reads were downloaded from the Short Read Archive (https://www.ncbi.nlm.nih.gov/sra, BioProject: in vitro SRP152081, in vivo SRP077418). All code and input/output files are available on github (https://github.com/jessstapley/QTL-mapping-Z.-tritici/). In brief, reads were trimmed and then mapped to 1 of 2 reference genomes (reads from 3D1 and 3D7 were mapped to the 3D7 reference genome, while reads from 1E4 and 1A5 were mapped to the 1A5 reference genome). Then, the number of reads mapping to each gene was counted using R::Rsubread (Liao *et al*. 2019), and we tested for differential gene expression (DGE) between strains, 3D7 vs 3D1 and 1A5 vs 1E4, using R::EdgeR (Robinson *et al*. 2010). DGE was tested using an exact negative binomial test, and the *P*-values were adjusted with Benjamini and Hochberg's algorithm. Only genes with log fold coverage of >2 and an adjusted *P*-value of <0.05 were considered significant.

### GO annotation

We took all the genes within the unique temperature stress QTL intervals and grouped them according to their trait/temperature combination—e.g. colony size–related traits at 10°C, or melanization-related traits at 27°C. GO enrichment analysis was performed following this tutorial for nonmodel species (https://archetypalecology.wordpress.com/2021/01/27/how-to-perform-kegg-and-go-enrichment-analysis-of-non-model-species-using-r/). GO annotations were retrieved from the annotation files described above, and we used the R package “topGo” (Alexa and Rahnenfuhrer 2023) to perform a Fisher’s test on the genes within each QTL interval following the associated guidelines. We used a *P*-value threshold of *P* < 0.05 and the *P*-value was not adjusted for multiple testing. All 3 ontologies—biological process, cellular component, and molecular function—were analyzed. The background set for GO analysis was the entire gene/transcript set for each reference genome (3D7 and 1A5). KEGG pathway analysis was not performed because *K* numbers can be found for only 34 and 49% of transcripts for 3D7 and 1A5, respectively.

### HSP enrichment in unique temperature QTL

HSPs are numerous (44 annotated in our reference genomes) and widely distributed across all core chromosomes in the genome. We tested whether the number of HSP genes within a unique temperature QTL interval (Obs_HSP_) was more than expected by chance using a simulation approach. For each unique QTL interval, we created 10,000 random QTL intervals of the same size and on the same chromosome. We counted the number of HSP genes in these random intervals, created a frequency distribution, and calculated the probability that a random QTL was equal to the number we observed (Obs_HSP_/10,000). If the probability was <0.05, then we concluded that the observed value was greater than expected by chance.

### Overlap with GWAS SNPs associated with climate

We investigated the overlap between genes within our intervals and those found in previous GWAS analyses. Earlier GWAS analyses used different reference genome annotations; hence, the approach we used to find overlaps between the TSQTL and the GWAS significant SNPs differed according to the comparison. For the traitGWAS (Dutta *et al*. 2023), many traits were measured, but we only consider associations with growth and melanization traits measured at 15°C, or relative growth/melanization between 15 and 22°C. The SNPs and their genomic positions were reported in 5 reference genomes (IPO323, 3D7, 3D1, 1A5, and 1E4), so we could directly compare gene IDs from our QTL intervals to the traitGWAS results. For the other 2 GWAS analyses (Feurtey *et al*. 2023; Miñana-Posada *et al*. 2025), IPO323 was the reference genome; thus, we had to convert IPO323 gene names to the orthologous positions in our parental reference genomes. In the geaGWAS, we used the list of SNPs having significant associations with bioclimatic variables associated with temperature and rainfall (for details, see Feurtey *et al*. 2023). For the adaptGWAS (Miñana-Posada *et al*. 2025), we used a list of SNPs with significant associations with traits measured at low temperatures only (12 and 15°C). We used custom R scripts to find overlaps between these genes/SNPs and our unique temperature stress QTL intervals (TQTL) and the geaGWAS and adaptGWAS.

## Results

### Effect of temperature on growth and melanization

**Fig. 1.**
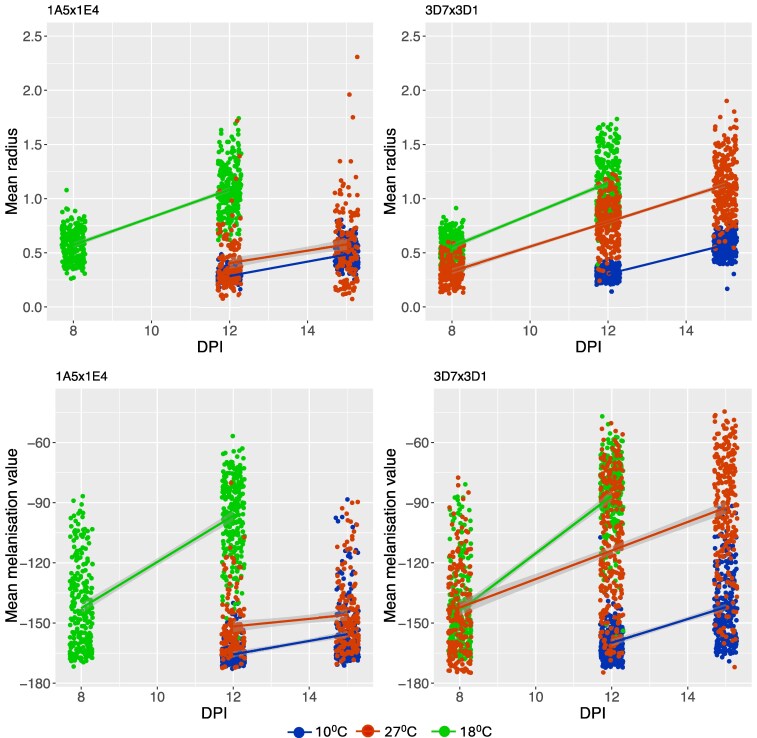
Mean colony radius and melanization at 3 temperatures plotted against dpi. Top panel: Colonies grown at high (27°C, red points) or low (10°C, blue points) temperatures had a smaller colony radius than those grown at 18°C, and were less melanized (lower panel). NB: Melanization value—inverse of the gray value.

A total of 209,258 and 194,536 colonies were phenotyped from the 1A5 × 1E4 and 3D7 × 3D1 crosses, respectively (see Supplementary Table 3 for details). Under both hot and cold stress, colony radius was reduced compared with the benign temperature (18°C) in both crosses (Fig. 1, Supplementary Table 3). The colony melanization was also lower under temperature stress, except at 8 dpi in 27°C in the 3D7 × 3D1 cross. We can only compare growth and melanization rate from 8 to 12 dpi between 27 and 18°C in the 3D7 × 3D1 cross. We found that growth rate and melanization rate were both significantly lower under heat stress (Supplementary Table 3). All pairwise correlations (Pearson, nonparametric) between independent measurements are provided in Supplementary Tables 4 and 5. We identified many correlations between independent measurements (Supplementary Tables 4 and 5), after excluding traits that are statistically or functionally coupled, including correlations between 2 time points of the same trait (i.e. radius at 8 and 12 dpi in the control), correlations between a rate and the corresponding time point measurements (i.e. gray value at 12 dpi and melanization rate in the control), or correlations between rates and tolerance measurements.

### QTL results

**Fig. 2.**
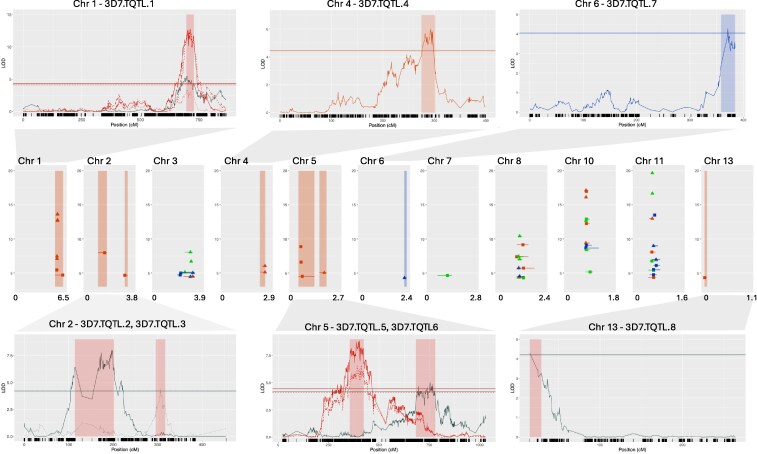
Center plot shows the genomic location of all significant QTL intervals and the peak LOD value for that interval for the 3D7 × 3D1 cross. The *y*-axis is LOD and *x*-axis is genomic position in Megabases. The shading indicates the unique temperature QTL intervals (3D7.TQTL), red for 27°C, blue for 10°C. Colors indicate whether the QTL were associated with traits measured in control (green, 18°C), low (blue, 10°C), or high (red, 27°C) temperature environments, and symbols indicate whether the trait is a colony size (circle) or melanization (triangle) related trait. For each TQTL, the detailed LOD profile is provided above or below the center plot, where the *y*-axis is LOD and the *x*-axis is chromosome position in centimorgans, and dark lines on the *x*-axis represent SNP positions. Some TQTL have multiple traits mapping to the overlapping interval. For plotting, we only included 1 time point trait (solid line), 1 tolerance trait (dashed line), and 1 rate trait (dotted). Line color relates to trait, red: colony size–related traits at 27°C, blue: colony size–related traits at 10°C, black: melanization-related trait at any temperature. Shading shows the 95% confidence interval of the QTL, the shading color relates to the temperature: red for 27°C, blue for 10°C.

**Fig. 3.**
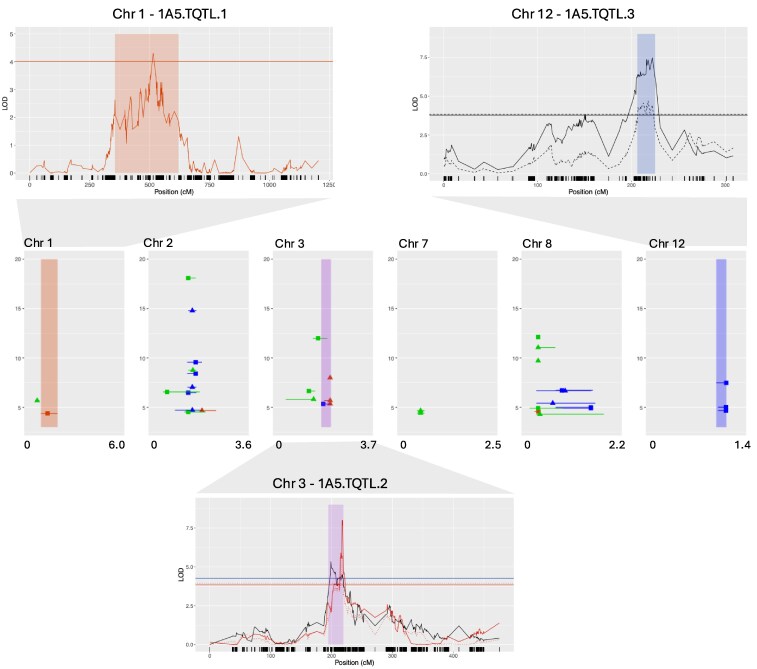
Center plot shows the genomic location of all significant QTL intervals and the peak LOD value for that interval for the 1A5 × 1E4 cross. The *y*-axis is LOD and *x*-axis is genomic position in Megabases. The shading indicates the unique temperature QTL intervals (1A5.TQTL), red for 27°C, blue for 10°C, purple for both. Colors indicate whether the QTL were associated with traits measured in control (green, 18°C), low (blue, 10°C), or high (red, 27°C) temperature environments, and symbols indicate whether the trait is a colony size–related (circle) or melanization-related (triangle) trait. For each TQTL, the detailed LOD profile is provided above or below the center plot, where the *y*-axis is LOD and *x*-axis is chromosome position in centimorgans, and dark lines on the *x*-axis represent SNP positions. Some TQTLs have multiple traits mapping to the overlapping interval. For plotting, we only included 1 time point trait (solid line), 1 tolerance trait (dashed line), and 1 rate trait (dotted). Line color relates to trait, red: colony size–related traits at 27°C, blue: colony size–related traits at 10°C, black: melanization-related trait at any temperature. Shading shows the 95% confidence interval of the QTL, the shading color relates to the temperature: red for 27°C, blue for 10°C, purple for both.

Parent and progeny isolates used in this study are from 2 crosses between Swiss strains and SNP genotype data for each set of progeny were obtained from a RAD sequence dataset previously produced in our lab (first described in Lendenmann *et al*. 2014). Linkage maps built using these SNPs were described previously (Stapley and McDonald 2023). After filtering and map building, 63,181 SNPs were available for analyzing the 3D7 × 3D1 cross and 32,806 SNPs were available for the 1A5 × 1E4 cross (Supplementary Table 2a). We found several QTL associated with growth and melanization traits (Table 2, Figs. 2 and 3). In the 3D7 × 3D1 cross, significant QTL were found for all but 1 trait (growth rate heat tolerance 8–12 dpi), and in the 1A5 × 1E4 cross, we found significant QTL for all but 4 traits (gray value in 27°C at 12 dpi, gray value in 27°C at 15 dpi, growth rate 10°C 12–15 dpi, melanization rate 27°C 12–15 dpi). We separated QTL into those that were uniquely associated with temperature stress from those that were shared across benign and temperature stress environments as described in the *Methods* section. In the 3D7 × 3D1 cross, 8 unique temperature QTL (3D7.TQTL) intervals were found (Table 3, Fig. 2), and in the 1A5 × E4 cross, 3 intervals (1A5.TQTL) were found (Table 3, Fig. 3). For full details of these intervals, see Supplementary Tables 6 and 7. Within unique temperature QTL intervals, potential candidate genes were identified by combining information from multiple sources, including gene function, sequence variation between parents, GO enrichment, RNAseq data, and the presence of SNPs identified in previous GWAS analyses (Dutta *et al*. 2023; Feurtey *et al*. 2023).

**Table 3.** Summary of the unique temperature QTL intervals, including the TQTL ID, associated chromosome, temperature stress (heat/cold/both), and the number of traits associated with this locus (M, melanization; S, colony size; B, both).

QTL ID	Chr	Temp	No. of traits (type)	Start	Stop	Interval	Max LOD	No. genes	No. Tgenes	Temp-genes	HSP enrich	GO enrich	geaGWAS	traitGWAS	adaptGWAS
3D7.TQTL.1	1	Heat	8 (B)	5189.86	6311.30	1121.44	13.15	391	5	2 × HSP, 1 × MAPKK Ssk2/Ssk22, 2 × C2H2	*P* = 0.27	19	47	1 (RCA)	5 (D2.12C, eAUC12C)
3D7.TQTL.2	2	Heat	1 (M)	1004.51	1626.25	621.74	8.05	250	7	2 × C2H2, 3 × HSP, 2 × PTP	*P* = 0.32	15	8	0	1 (x75.12C)
3D7.TQTL.3	2	Heat	1 (M)	3169.15	3428.44	259.30	4.39	67	1	1 × cAMP	NA	5	2	0	0
3D7.TQTL.4	4	Heat	3 (S)	2455.95	2646.71	190.77	6.12	124	1	1 × HSP	*P* = 0.33	3	18	1 (M15C)	0
3D7.TQTL.5	5	Heat	3 (S)	399.93	1237.36	837.43	8.95	332	3	2 × HSP, 1 × PTP	*P* = 0.04	13	37	0	0
3D7.TQTL.6	5	Heat	1 (M)	1661.50	2062.60	401.10	5.03	21	6	4 × HSP, 3 × C2H2	*P* = 0.06	8	3	**0**	0
3D7.TQTL.7	6	Cold	1 (S)	2260.91	2383.12	122.21	4.29	40	0		NA	4	1	0	0
3D7.TQTL.8	13	Cold	1 (M)	23.95	82.58	58.62	4.39	124	0		NA	1	1	0	0
															
1A5.TQTL.1	1	Heat	1 (S)	1037.31	1949.98	912.67	4.39	439	5	1 × Opy2, 4 × HSP, 1 × MAP, 1 × PTP, 1 × cAMP(PKA)	*P* = 0.08	19	23	0	0
1A5.TQTL.2	3	Both	4 (B)	1843.71	2251.85	408.13	8.00	114	2	1 × Sho1, 1 × PTP	NA	3	7	1 (RCA)	0
1A5.TQTL.3	12	Cold	3 (M)	1070.06	1234.96	164.90	7.48	61	2	1 × C2H2, 1 × PTP	NA	2	3	0	0

For the largest significant interval mapping to this locus, data include start and stop positions and interval size in kilobases, maximum LOD, number of genes, number of Temp-genes (Tgenes), Temp-gene names, the *P*-value indicating whether the interval contained more HSPs than expected by chance, the number of genes with enriched GO annotations, the number of genes overlapping with the traitGWAS-SNPs, the geaGWAS-SNPs, or the adaptGWAS.

RCA, relative colony area 14–22C; M15C, melanin 15C; D2.12C, day2_12C; x75.12C, xq75total_12C.

### GO annotations and HSP enrichment in TQTL

We grouped all genes from TQTL intervals according to the stress (heat/cold) and the trait (colony size/melanization) to create gene sets. In both crosses, we had 3 gene sets for analysis, and the results are provided in Supplementary Tables 8 and 9. The largest enriched GO annotation for 3D7 × 3D1, in terms of the highest number of significant genes, was “zinc ion binding” (GO:0008270). The interval 3D7.TQTL.1 had 12 genes with this GO annotation, and the interval 3D7.TQTL.2 had 13 genes with this annotation. In the 1A5 × 1E4 cross, the 2 GO annotations with the highest number of significant terms were “protein phosphorylation” (GO:0006468) and “protein kinase activity” (GO:0004672). Fourteen genes had these GO annotations in the interval 1A5.TQTL.1. One interval, 3D7.TQTL.5, was significantly enriched for HSPs (*P* = 0.04, Table 3). Two other TQTL intervals, 1 in each cross (3D7.TQTL.6 and 1A5.TQTL.1), had 4 HSPs. The probability of observing 4 HSPs in an interval on each of those 2 respective chromosomes was 0.06 and 0.08 (Table 3); thus, although it was uncommon (*P* < 0.10), HSP enrichment was not significant at *P* < 0.05 in these 2 QTLs.

### Most promising candidate genes associated with temperature stress in 3D7 × 3D1

The 3D7.TQTL interval with the highest LOD was on chromosome 1 (3D7.TQTL.1), and it was associated with 6 colony size–related traits (colony radius in 27°C at all time points, colony radius tolerance at 8 and 12 dpi and growth rate between 8 and 12 dpi) and 2 melanization-related traits (mean gray value at 27°C at 12 and 15 dpi). The colony size–related QTL had a higher LOD (>10) than the melanization traits (Fig. 2). The largest significant interval spanned 1,121 kb and contained 391 genes. Of these 391 genes, 46 contained geaGWAS-SNPs, 1 traitGWAS-SNP, and 19 had enriched GO terms (Table 3, Supplementary Table 10). We provide a summary of several genes of interest (Table 4) and highlight next the 4 most promising candidate genes in this interval.

**Table 4.** Details connected with the most promising candidate genes found within TQTL intervals.

TQTL ID	Chr	ZT3D7 Gene ID	ZT3D7 Prot.ln	geaGWAS-SNP	traitGWAS-SNP	adaptGWAS-SNP	GO annotation enriched	ZT3D1 Gene ID	ZT3D1 Prot.ln	Protein sim (3D7, 3D1)	High-impact var.	DEG in vitro	DEG in vivo	IPO323.geneID	IPO323 Prot.ln	Protein sim (IPO, 3D7)	Protein sim (IPO, 3D1)	Predicted domain
3D7.TQTL.1	1	ZT3D7_G1860	1654	NA	NA	NA	No	ZT3D1_G1822	1618	91.88	3	Yes	Yes	ZtIPO323_019890	1688	94.92	93.06	Histidine kinase/HSP90-like ATPase superfamily
3D7.TQTL.1	1	ZT3D7_G1950	810	BIO6	NA	NA	Yes	ZT3D1_G1910	867	92.96	0	Yes	Yes	ZtIPO323_020880	300	100	87	Probable general stress response protein Whi2 Alcohol dehydrogenase GroES-like domain, Zinc-binding dehydrogenase
														ZtIPO323_020890	360	99	99	Alcohol dehydrogenase
3D7.TQTL.1	1	ZT3D7_G1951	1385	NA	NA	NA	No	ZT3D1_G1911	1419	99.93	0	Not sig.	NA	ZtIPO323_020900	1419	99.86	99.93	Protein kinase domain, MAPKKK Ssk2/Ssk22
3D7.TQTL.1	1	ZT3D7_G2013	581	NA	RCA	NA	No	ZT3D1_G1975		88.66	1	Yes	Not sig.	ZtIPO323_021600	581			No predicted function
3D7.TQTL.1	1	ZT3D7_G2052	520	NA	NA	eAUC12C	No	ZT3D1_G2016	520	99.23	0	Not sig.	Not sig.	ZtIPO323_022050	520	99.42	98.65	Ammonium transporter family
3D7.TQTL.4	4	ZT3D7_G5373	449	NA	M15C	NA	No	ZT3D1_G5304	449	99.78	0	Not sig.	Yes	ZtIPO323_058350	362	100	99.72	MOSC N-terminal beta barrel domain, MOSC domain

TQTL ID	Chr	ZT3D7 Gene ID	ZT1A5 Prot.ln	geaGWAS-SNP	traitGWAS-SNP	adaptGWAS-SNP	GO annotation enriched	ZT1E4 Gene ID	ZT1E4 Prot.ln	Protein sim (1A5, 1E4)	High-impact var.	DEG in vitro	DEG in vivo	IPO323.geneID	IPO323 Prot.ln	Protein sim (IPO, 1A5)	Protein sim (IPO, 1A4)	Predicted domain
1A5.TQTL.1	1	ZT1A5_G381	1547	NA	NA	NA	No	ZT1E4_G375	1547	99.55	1	Not sig.	Not sig.	ZtIPO323_004060	1513	97.29	97.48	Histidine kinase/HSP90-like ATPase superfamily
1A5.TQTL.1	1	ZT1A5_G420	414	NA	NA	NA	Yes	ZT1E4_G414	414	100	0	Not sig.	Not sig.	ZtIPO323_004450	414	100	100	Protein kinase domain, mitogen-activated protein (MAP) kinase
1A5.TQTL.1	1	ZT1A5_G473	185	NA	NA	NA	No	ZT1E4_G467	185	96.22	0	Not sig.	Not sig.	ZtIPO323_005050	187	95.14	96.24	PTP, Low molecular weight phosphotyrosine protein phosphatase
1A5.TQTL.1	1	ZT1A5_G491	442	BIO5	NA	NA	No	ZT1E4_G485	453	90.27	1	Not sig.	Not sig.	ZtIPO323_005250	459	91.94	88.06	WD40-repeat-containing domain superfamily
1A5.TQTL.1	1	ZT1A5_G492	476	BIO5, BIO10	NA	NA	No	ZT1E4_G486	476	99.79	0	Not sig.	Not sig.	ZtIPO323_005260	476	99.58	99.79	Opy2 protein
1A5.TQTL.1	1	ZT1A5_G634	1178	BIO3	NA	NA	No	ZT1A5_G632	1176	97.28	0	Not sig.	Not sig.	ZtIPO323_006800	1176	97.87	97.88	Histidine kinase/HSP90-like ATPase superfamily
1A5.TQTL.2	3	ZT1A5_G4068	329	NA	NA	NA	No	ZT1E4_G4135	329	100	0	Not sig.	Not sig.	ZtIPO323_044480	329	100	100	SH3 domain, Sho1, SH3 domain, high-osmolarity signaling protein Sho1, SH3-like domain superfamily
1A5.TQTL.2	3	ZT1A5_G4089	549	NA	RCA	NA	No	ZT1E4_G4154	549	100	0	Yes	Not sig.	ZtIPO323_044730	549	100	100	Cytochrome P450
1A5.TQTL.3	12	ZT1A5_G11001	779	NA	NA	NA	No	ZT1E4_G11018	779	99.49	0	Yes	Not sig.	ZtIPO323_120430	780	99.48	99.74	Zinc finger, C2H2 type
1A5.TQTL.3	12	ZT1A5_G11023	355	NA	NA	NA	No	ZT1E4_G11044	355	100	0	Yes	Yes	ZtIPO323_120710	390	100	100	Tyrosine phosphatase family (PTP)

This includes: cross, TQTL ID, chromosome (Chr), gene ID, protein length (Prot.ln), the bioclimatic variable associated with any GWAS-SNP (geaGWAS), the trait association with GWAS-SNP (traitGWAS), association with SNP in thermal adaption GWAS (adapGWAS), whether the gene had an enriched GO annotation (Protein Sim.) between the parents, whether the gene was differentially expressed in vitro or in vivo, IPO323 gene ID, IPO323 protein length and protein similarity between IPO323 and parents, and relevant protein family domain within the gene.

The first gene encodes an HSP90-like ATPase superfamily domain (ZT3D7_G1860) that we consider a promising candidate because it varied between the 2 parents, including 3 high-impact variants (2 “frameshift variants,” 1 “stop gained”) likely to affect protein function. Under benign temperature the gene is differentially expressed in vitro and in vivo (Table 4). This gene was in the high LOD peak associated with colony size–related traits.

The second promising candidate gene is a “probable general stress response protein *Whi2*.” This gene is not annotated in the 3D7 and 3D1 genomes, but it is annotated in the most recent IPO323 annotation (gene ID: ZtIPO323_020880) and is orthologous with part of ZT3D7_G1950, which has an enriched GO term (zinc ion binding, GO:0008270) and a geaGWAS-SNP for minimum temperature of coldest month (BIO6; Table 4). The gene ZtIPO323_020880, predicted to encode *Whi2* has 100% protein conservation with part of ZT3D7_G1950, but only 87% protein conservation with part of ZT3D1_G1910 (Table 4). Based on the 3D7 genome annotation, this gene contains an alcohol dehydrogenase GroES-like domain (GroES is an HSP for *Escherichia coli*) and a zinc-binding dehydrogenase domain. After blasting the alcohol dehydrogenase protein (ZtIPO323_020890) against the 3D7 and 3D1 genomes, we found only one mismatch in ZT3D1_G1910 and 2 in ZT3D7_G1950 among the 360 amino acids encoded by this gene. The genomic region containing ZT3D7_G1950 and ZT3D1_G1910 needs to be reannotated in these respective reference genomes.

The third candidate (ZT3D7_G1951) is immediately adjacent to ZT3D7_G1950 and ZT3D1_G1910 and is a MAPKKK *SSk2/Ssk22* (*ZtSsk2*) gene, which is part of the HOG1 pathway (Dunayevich *et al*. 2018). *ZtSsk2* is homologous across the 3 relevant genomes (ZT3D7_G1951, ZT3D1_G1911, and ZtIPO323_020900) with little protein variation, only one amino acid difference between ZT3D1_G1911 and ZT3D7_G1951 (alanine–valine), and there are no significant differences in gene expression under benign temperatures (Table 4).

The fourth promising candidate gene in this interval is ZT3D7_G2052, which encodes an ammonium transport family domain. This gene has multiple adaptGWAS-SNPs and the protein varies between the parents, with no high-impact SNPs, but 4 SNPs predicted to have moderate effects (https://github.com/jessstapley/QTL-mapping-Z.-tritici/). The gene was upregulated in 3D7 in vitro growing on MM.

### Most promising candidate genes associated with temperature stress in 1A5 × 1E4

On chromosome 1, the 1A5.TQTL.1 was associated with the binary trait colony growth at 27°C. This interval was large and contained 439 genes (Table 3, Supplementary Table 11), including 19 genes with enriched GO annotations and 22 genes with a geaGWAS-SNP. This interval contains several promising candidates (Table 4), including the HOG1 pathway gene highlighted in the geaGWAS, *Opy2* (ZT1A5_G492). The *Opy2* geaGWAS-SNP was associated with the maximum temperature of the warmest month (BIO5, −log(*P*-value) = 7.65) and mean temperature of warmest quarter (BIO10, −log(*P*-value) = 7.60). There is 1 amino acid difference between the parents (arginine–serine). The second promising candidate is an HSP gene with a geaGWAS-SNP associated with isothermality (BIO3: mean diurnal temperature range/temperature annual range) (ZT1A5_G634). This protein, which is 1,176 amino acids long, has 32 amino acids different between the parents and 37 moderate-effect variants (missense variants). The second unique temperature QTL 1A5.TQTL.2 for this cross was on chromosome 3. It was associated with both hot and cold stress and contained the HOG1 signaling protein, *Sho1* (ZT1A5_G4068; Tables 3 and 4) as well as a cytochrome P450 gene with a traitGWAS-SNP (ZT1A5_G4089). The protein products of these genes were conserved between the parents, and with the IPO323v2 annotation. No significant differences in gene expression between parents were found for the Sho1 gene in vitro or in vivo, but the cytochrome p450 gene was upregulated in vitro in the 1E4 parent (Supplementary Table 11).

On chromosome 12, there was a cold-stress-QTL interval (1A5.TQTL.3) associated with 3 melanization traits (melanization rate 10°C 12–15 dpi, gray value in 10°C at 15 dpi, gray value in 10°C at 12 dpi). This interval contained 62 genes and overlapped, by a single gene, with a QTL previously identified for the morphological transition from yeast-like growth to hyphal growth at high temperature (Francisco *et al*. 2023; Supplementary Table 11). Within the interval, there were 2 promising candidate genes (Table 4), including ZT1A5_G11001 which has a zinc finger C2H2-type domain, and ZT1A5_G11023, which has a protein-tyrosine phosphatase-like (PTP) domain. Both genes showed differential expression between the parents (Table 4). The zinc finger C2H2-type gene produced a different protein in the parents and the IPO323v2 ortholog is predicted to encode the transcriptional regulator CRZ1. CRZ1 was shown to be involved in CWI and melanization in *Alternaria alternata* (Yang *et al*. 2022) and heat-shock response in pathogenic fungi (Roy *et al*. 2021).

## Discussion

We identified 13 and 11 distinct QTL intervals associated with growth and melanization in the 3D7 × 3D1 and 1A5 × 1E4 crosses, respectively (Figs. 2 and 3). Among these, we identified 11 QTL intervals uniquely associated with temperature stress (TQTL), with 8 such intervals in the 3D7 × 3D1 cross and 3 in the 1A5 × 1E4 cross (Figs. 2 and 3, Table 3). Based on information gathered from multiple sources, including previous GWAS analyses and functional annotations, we highlight several promising candidate genes for temperature responses, including HSP90 genes, a *Whi2* gene, a zinc-dependent alcohol dehydrogenase, an ammonium transporter, and 2 HOG pathway genes *SSk2* and *Opy2*. HSPs were common in the TQTL intervals. There was some evidence of enrichment (Table 3), 3D7.TQTL.5 had significantly (*P* = 0.04) more HSPs than the number of HSPs observed in 10,000 random QTL intervals. Two intervals had 4 HSPs, and although this was not significantly (*P* < 0.05) greater than the number of HSPs observed in 10,000 random QTL intervals, it was uncommon, occurring in only 6 and 8% of these randomly generated intervals for 3D7.TQTL.6 and 1A5.TQTL.1, respectively. In a previous QTL study using the same isolates grown in vitro under cool-temperature (15°C) stress, no enrichment of HSPs was found (Lendenmann *et al*. 2016), but our new analysis that included a broader range of temperatures suggests that HSPs may contribute to responses to temperature.

### Candidates for temperature stress response identified in the 3D1 × 3D7 cross

HSPs form an integral part of the general stress response in many organisms (Richter *et al*. 2010). We highlighted an HSP90-like ATPase superfamily gene (ZT3D7_G1860) as a promising candidate. HSP90 is the most common form of HSP in yeast and the most common predicted HSPs in our reference genomes (31 in 3D7, 32 in 1A5). HSP90 interacts with hundreds of proteins under both stressful and stress-free environments (Crunden and Diezmann 2021). In *Candida albicans*, HSP90s regulate the transition between yeast and filamentous forms as well as virulence (Robbins and Cowen 2023). The 3D7.TQTL.6 interval had 4 HSPs, 2 of which were HSP70 genes (ZT3D7_G6242, ZT3D7_G6240). HSP70s are less common in the genome (11 in 3D7 and 12 in 1A5), but are one of the most conserved HSPs. They are involved in protein folding, both de novo folding and refolding of aggregated proteins, and prevent aggregation of denatured proteins under stress (Richter *et al*. 2010). In *Z. tritici*, expression of 4 HSP70 genes was upregulated at the transition stage from biotrophy to necrotrophy (7–11 dpi) in planta (Palma-Guerrero *et al*. 2016). One of these, Mycgr3G72449 (Zt09_model_5_00606), is the ortholog of ZT3D7_G6242 (100% similarity at the protein level) which is in the 3D7.TQTL.6 interval. Another notable feature of the 2 HSP70 genes in the 3D7.TQTL.6 interval is that they are very close, separated by only a single gene, which is a zinc finger C2H2-type gene.

We identified most of the candidate genes in the high-temperature-response QTL on chromosome 1 (3D7.TQTL.1; Table 4). This QTL contained 47 genes with SNPs significantly associated with climatic variables (Feurtey *et al*. 2023) and was associated with growth at cooler temperatures [relative colony area between 15 and 22°C (Dutta *et al*. 2023), and growth at 12°C (Miñana-Posada *et al*. 2025)]. In the adaptGWAS, 1 gene contained 7 significant SNPs associated with 12°C growth across a panel of 411 isolates (Miñana-Posada *et al*. 2025). This gene encodes an ammonium transporter-like protein (Table 4). In contrast to the previous QTL and GWAS studies, our QTL was associated with growth and melanization under high temperature (27°C) but not cold temperature (10°C). If these candidate genes are related to a response to temperature, then our results, combined with the previous studies, suggest that they are affecting a general temperature response rather than a response limited to either high or low temperatures.

The other genes we highlight as promising candidates are 2 adjacent genes in 3D7.TQTL.1. One has orthology with a probable general stress response protein *Whi2 (*ZT3D7_G1950) and the other gene encodes a MAPKKK *SSk2/Ssk22* domain (ZT3D7_G1951). ZT3D7_G1950 is a potential candidate because it has an enriched GO term (GO:0008270, zinc ion binding), has a geaGWAS-SNP for minimum temperature of coldest month (Table 4), and was differentially expressed between the parents—the gene was upregulated in 3D1 compared with 3D7 in MM and on Drifter at 28 dpi (Supplementary Table 10). There is no 1-to-1 homology of ZT3D7_G1950 with the most recent IPO323 genome and alignment of this region between the 2 parents (3D7 and 3D1) and the IPO323 genome suggests the annotation in 3D7 and 3D1 needs updating. Based on DNA and protein similarity with IPO323, there is a gene encoding *Whi2* and a zinc-dependent ADH at this position in 3D7 and 3D1. WHI2 (Whiskey2) is a general stress response factor that can influence the expression of stress-associated genes, nutrient sensing, and the transition from biotrophic infection to necrotrophic infection in fungi (Kaida *et al*. 2002; Shuai *et al*. 2022). Whether *Whi2* affects a response to temperature in pathogenic fungi has, to our knowledge, not been tested, but in the rice false smut pathogen *Ustilaginoidea virens*, a *UvWhi2* knockout had increased sensitivity to oxidative stress and cell-wall stress (Shuai *et al*. 2022), both of which can be induced by heat stress. ADHs are important in growth and metabolism under both aerobic and anaerobic conditions and are known to affect virulence in many fungal genera (Gutiérrez-Corona *et al*. 2023). The roles played by ADHs under temperature stress in pathogenic fungi remain unclear; however, ADHs were shown to be involved in cold-stress tolerance in plants (Christie *et al*. 1991) and the oleaginous alga *Nannochloropsis salina* (Lim *et al*. 2023). ADHs also play important roles in tolerance to oxidative stress (Gutiérrez-Corona *et al*. 2023), which can be induced by temperature stress (Xiao *et al*. 2022). The other potential candidate, *SSk2*, is involved in the HOG1 pathway and was already shown to play a role in heat stress response in yeast (Dunayevich *et al*. 2018). In *Z. tritici*, loss-of-function mutations in *Ssk2* led to greater sensitivity to osmotic and oxidative stress, loss of virulence on a susceptible host (cv. Riband), and increased resistance to fungicides (Blyth *et al*. 2023). It would be worthwhile to test the responses of *Ssk2* mutants to temperature stress.

### Candidates for temperature stress response in the 1A5 × 1E4 cross

A notable feature of this cross was that the 1E4 parent and approximately half of the offspring in this cross did not grow under heat stress (27°C). We identified a QTL associated with this binary trait (growth/no-growth at 27°C), 1A5.TQTL.1. Within this QTL interval, there were many Temp-genes, many genes with geaGWAS-SNPs and 1 gene with a traitGWAS-SNP. A notable gene with a geaGWAS-SNP is *Opy2*. *Opy2* has been shown to be involved in responses to stress and affects virulence in fungi. In *S*accharomyces *cerevisiae*, *Opy2* transmembrane protein is involved in the *Sho1* branch of the HOG1 pathway during osmotic stress (Smith *et al*. 2010). It was also shown to play a role in the filamentous growth pathway in response to glucose limitation (Karunanithi and Cullen 2012). In *C. albicans*, *CaOpy2* does not play a role in adaptation to osmotic stress, but it is required for *Cek1* phosphorylation and plays an essential role in cell-wall stress (Herrero De Dios *et al*. 2013). *Opy2* was shown to affect virulence in *Metarhizium robertsii* (Guo *et al*. 2017) and *Magnaporthe oryzae* (Cai *et al*. 2022). The protein products of genes encoding *Opy2* in our 2 parents (ZT1A5_G492 and ZT1E4_G486) were very similar, with only a single amino acid difference (arginine–serine), but *Opy2* expression did not differ between the parents on different media or during infection at benign temperatures (Supplementary Table 11). In a previous study searching for QTL associated with osmotic stress using the same isolates, 1 osmotic stress QTL on chromosome 1 was found, and the *Opy2* gene was present in that interval (Stapley and McDonald 2023). The QTL was associated with 3 traits (colony radius KCl tolerance at 12 dpi, growth rate KCl tolerance, and colony radius in KCl at 12 dpi) but had a rather low LOD (4.03–4.80). The largest interval of these 3 QTL spanned 5,583 kb and contained over 2,000 genes. Because of the relatively low LOD and large interval, we did not consider possible candidates in this interval in the previous study, but focused instead on a smaller interval that had a larger LOD (Stapley and McDonald 2023). Taking into account the finding that *Opy2* is present in independent QTL for osmotic and temperature stress, we speculate that heat stress may trigger changes in turgor pressure that can be sensed by the osmosensor *Opy2* and trigger the CWI pathway, as was found in yeast (Smith *et al*. 2010; Dunayevich *et al*. 2018).

The 1A5.TQTL.3 on chromosome 12 associated with cold stress partially overlapped with a previously mapped QTL for a heat-induced blastospore-to-hyphae/chlamydospore transition (Francisco *et al*. 2023). The previous study functionally validated 2 genes that regulate the blastospore-to-hyphae/chlamydospore transition in response to heat stress in *Z. tritici*, including a novel transcription factor named *ZtMsr1* and a protein phosphatase, *ZtYvh1*. The overlapping region between 1A5.TQTL.3 and the previous interval is small and only contains 1 gene (ZT1A5_G11033), not the 2 genes functionally annotated in the previous study. Our interpretation in this case is that the 2 traits involve different genes even though the QTLs showed a small overlap.

### QTL intervals associated with traits measured in stressful and benign temperature environments

We found a QTL on chromosome 10 that was associated with colony size and melanization traits measured in both cold stress and benign environments, as well as colony radius heat tolerance. Because there is an overlap between the intervals identified in stressful and benign environments, this is not a unique temperature stress QTL. However, it is worth mentioning because it overlaps with a QTL containing a candidate gene (*Pbs2*) for cold sensitivity previously identified in the 3D7 × 3D1 cross (Lendenmann *et al*. 2016). *Pbs2* is upstream of HOG1 and is downstream of *SSk2* and *Opy2*. In the previous QTL study, colonies were grown at 15 and 22°C, thus under a milder cold stress compared with our current study. A QTL associated with oxidative stress tolerance was also found on chromosome 10 in a previous study (Zhong *et al*. 2021); however, that QTL does not overlap with the QTL we identified in this study.

Another notable QTL interval is that on chromosome 11. This was previously found to be associated with melanization and colony size traits in stressful and benign environments (Lendenmann *et al*. 2014; Stapley and McDonald 2023) in the 3D7 × 3D1 cross. Regulation of the *Zmr1* transcription factor in this interval was shown to affect melanin production and is thought to explain most of the phenotypic variance associated with this QTL (Krishnan *et al*. 2018). We again found that multiple melanization- and growth-related traits measured under hot and cold stress were associated with this QTL interval (Fig. 2, Supplementary Table 6) and believe that the previous interpretations also apply in these cases.

In the 1A5 × 1E4 cross, we found a large QTL interval on chromosome 8 that included 11 traits, including colony size and melanization traits under temperature stress and benign temperature environments (Supplementary Table 7). The same QTL was found in previous studies mapping QTL for oxidative (Zhong *et al*. 2021) and osmotic stress (Stapley and McDonald 2023), and similar to the results of this study, the QTL was associated with traits measured in both benign and stressful environments. This genomic region likely contains genes that affect intrinsic growth of colonies under a wide range of environments and/or genes with pleiotropic effects.

### Notable temperature stress genes identified via GO annotation enrichment

A notable GO annotation was the zinc ion binding (GO:0008270) term. Many genes in unique temperature QTL had this annotation, and it was enriched amongst the gene set associated with melanization-related traits under heat stress. Zinc finger proteins are one of the largest transcription factor families in eukaryotes (Ding *et al*. 2022). Genes with this annotation encode many proteins, including C2H2-type zinc-finger proteins that are in our list of Temp-genes, and they are considered master regulators of stress response in mushrooms (Hou *et al*. 2020) and other eukaryotes (MacPherson *et al*. 2006). Genes encoding fungal Zn(2)-Cys(6) binuclear cluster proteins, which bind 2 zinc atoms with a DNA-binding domain consisting of 6 cysteine residues, also have this GO annotation, which is found over 100 times in our reference genomes (3D7:102, 1A5:125). In mushrooms, these have been shown to be differentially expressed under cold and heat stress (Ding *et al*. 2022). In *Z. tritici*, the Zn(2)-Cys(6) binuclear cluster domain gene ZtMsr1 is involved in the blastospore-to-hyphae/chlamydospore transition that is triggered by heat stress. Deletion of ZtMsr1 in the 1A5 strain activated hyphal growth, whereas the insertion of the 1A5-ZtMsr1 allele into the 1E4 strain (in which ZtMsr1 is naturally disrupted by a TE), induced chlamydospore formation at 27°C (Francisco *et al*. 2023). This example illustrates how zinc finger proteins can influence important fungal traits under temperature stress.

## Supplementary Material

jkaf094_Supplementary_Data

## Data Availability

All data are available at ETH Data repositories: for SNP data, DOI: 10.3929/ethz-b-000550424, and for phenotype data, DOI: 10.3929/ethz-b-000732068. All code and details of the analysis are available at https://github.com/jessstapley/QTL-mapping-Z.-tritici. Supplemental material available at G3 online.
