# Supplementary material for: Mapping genomic regions associated with temperature stress in the wheat pathogen Zymoseptoria tritici
Source: G3 (Bethesda). 2025 Apr 26;15(6):jkaf094. doi: 10.1093/g3journal/jkaf094 (PMC12135003; doi:10.1093/g3journal/jkaf094)
Supplement: jkaf094_Supplementary_Data [file jkaf094_supplementary_data.zip › Supplemental_Table_Legends_G3-2025-405813.docx]

**Supplemental Table Legends**

**Table S1.** A list of known temperature-response genes and their copy number in the parental reference genomes.

**Table S2.** Linkage map summary.

**Table S3.** Summary of phenotypic data. a) Number of isoales and colonies per treatment. b) comparison of trait values between 10^0^C and 18^0^C. c) comparison of trait values between 18^0^C and 27^0^C.

**Table S4.** All pairwise correlations betweent traits in 3D7x3D1 cross.

**Table S5.** All pairwise correlations betweent traits in 1A5x1E4 cross.

**Table S6.** All signfiicant QTL identified in the 3D7x3D1 cross.

**Table S7.** All signfiicant QTL identified in the 1A5x1E4 cross.

**Table S8.** Results of the GO enrichment analysis for 3D7x3D1 cross.

**Table S9.** Results of the GO enrichment analysis for 1A5x1E4 cross.

**Table S10.** Information on all genes in the unique temperature QTL in 3D7x3D1 cross.

**Table S11.** Information on all genes in the unique temperature QTL in 1A5x1E4 cross.
